# Supplementary material for: Comparative genomics provides new insights into the diversity, physiology, and sexuality of the only industrially exploited tremellomycete: Phaffia rhodozyma
Source: BMC Genomics. 2016 Nov 9;17:901. doi: 10.1186/s12864-016-3244-7 (PMC5103461; doi:10.1186/s12864-016-3244-7)
Supplement: Additional file 6: — List of orphan genes with links to PFAM (related to Additional file 1: Table S1). (ZIP 1428 kb) [file 12864_2016_3244_MOESM6_ESM.zip › PFAM_UNIQUE/G04588_P.html]

#                                                                            --- full sequence --- -------------- this domain -------------   hmm coord   ali coord   env coord
  
# target name        accession   tlen query name           accession   qlen   E-value  score  bias   #  of  c-Evalue  i-Evalue  score  bias  from    to  from    to  from    to  acc description of target
  
#------------------- ---------- ----- -------------------- ---------- ----- --------- ------ ----- --- --- --------- --------- ------ ----- ----- ----- ----- ----- ----- ----- ---- ---------------------
  
G04588\_P             -            219 ABM                  PF03992.11    78      0.02   13.6   0.0   1   1   1.7e-05     0.035   12.8   0.0     2    49   158   205   157   212 0.86 - Antibiotic biosynthesis monooxygenase  
G04588\_P             -            219 Mus7                 PF09462.5    614     0.047   11.2   0.2   1   1   2.8e-05     0.055   11.0   0.2   180   220    79   121    33   137 0.83 - Mus7/MMS22 family  
G04588\_P             -            219 Sorting\_nexin        PF03700.8    129      0.14   10.9   3.1   1   2   8.6e-05      0.52    9.1   0.1    44    88    48    93    15    97 0.81 - Sorting nexin, N-terminal domain  
G04588\_P             -            219 Sorting\_nexin        PF03700.8    129      0.14   10.9   3.1   2   2     0.013        76    2.1   0.7    20    59   113   151    95   159 0.70 - Sorting nexin, N-terminal domain
